# SESN2 Knockdown Increases Betulinic Acid-Induced Radiosensitivity of Hypoxic Breast Cancer Cells

**DOI:** 10.3390/cells12010177

**Published:** 2022-12-31

**Authors:** Antje Güttler, Claus Weinholdt, Elisabeth Ruff, Judith Reidt, Elisa Darnstaedt, Alicia Wildemann, Marina Petrenko, Jacqueline Keßler, Matthias Kappler, Ivo Grosse, Dirk Vordermark, Matthias Bache

**Affiliations:** 1 Department of Radiotherapy, Martin Luther University Halle-Wittenberg, Ernst-Grube-Str. 40, 06120 Halle, Germany; 2 Institute of Computer Science, Martin Luther University Halle-Wittenberg, Von-Seckendorff-Platz 1, 06120 Halle, Germany; 3 Department of Oral and Maxillofacial Plastic Surgery, Martin Luther University Halle-Wittenberg, Ernst-Grube-Str. 40, 06120 Halle, Germany; 4 German Centre for Integrative Biodiversity Research (iDiv) Halle-Jena-Leipzig, Puschstrasse 4, 04103 Leipzig, Germany

**Keywords:** betulinic acid, sestrin-2, knockdown, radiosensitivity, autophagy, breast cancer

## Abstract

Betulinic acid (BA) is a natural compound well known for its anti-inflammatory, anti-viral, anti-bacterial, anti-malarial effects and anti-tumor properties. Its enhanced cytotoxicity in tumor cells and induction of cell death in various cancer entities qualifies BA as an interesting candidate for novel treatment concepts. Our analyses showed enhanced cytotoxicity and radiosensitization under hypoxic conditions in human breast cancer cells. So far, the underlying mechanisms are unknown. Therefore, we investigated the BA-treated human breast cancer cell lines MDA-MB-231 and MCF-7 under normoxic and hypoxic conditions based on microarray technology. Hypoxia and BA regulated a variety of genes in both breast cancer cell lines. KEGG pathway analysis identified an enrichment of the p53 pathway in MCF-7 cells (wtp53) under hypoxia. In MDA-MB-231 cells (mtp53) an additional BA incubation was required to activate the p53 signaling pathway. Fourteen down-regulated and up-regulated genes of the p53 pathway were selected for further validation via qRT-PCR in a panel of five breast cancer cell lines. The stress-induced gene Sestrin-2 (*SESN2*) was identified as one of the most strongly up-regulated genes after BA treatment. Knockdown of *SESN2* enhanced BA-induced ROS production, DNA damage, radiosensitivity and reduced autophagy in breast cancer cells. Our results identified SESN2 as an important target to enhance the radiobiological and anti-tumor effects of BA on breast cancer cells.

## 1. Introduction

Since 2020, breast cancer is the most common cancer in the world: 2.3 million new cases representing 11.7% of all cancer cases worldwide [1]. The options for breast cancer treatment range from surgery and radiation to chemo-, immuno- and hormone therapy and are very effective if the disease has been detected at an early stage. Nevertheless, in women it is the leading cause of cancer-related death. Patients with breast cancer in advanced stages or triple-negative breast cancer (TNBC) have a particularly poor prognosis [2]. Tumor hypoxia is known to result in therapy resistance, especially to radio- and chemotherapy, and is associated with an unfavorable prognosis in breast cancer [3]. HIF-1α is one of the major regulators of tumor hypoxia and is overexpressed in many tumors, including breast cancer and also associated with poor prognosis. Therapeutic approaches to overcome hypoxia using HIF inhibitors showed inhibited tumor growth and metastasis, and tumor regression in combination with other chemotherapeutics, in mice [4]. However, it is pivotal for further research to improve therapeutic options and prognosis especially for TNBC patients. Hence, natural compounds provide an opportunity for alternative focuses of research.

Betulinic acid (BA), a pentacyclic triterpene, is a natural product from birch bark with well-described anti-tumor effects in many cell lines of different tumor entities, such as melanoma, colorectal, prostate, lung and breast cancer, as well as in xenograft mouse models [5,6]. One remarkable property of BA is the increased cytotoxicity detected in tumor cells compared to normal tissue and under hypoxic compared to normoxic conditions [7,8]. Especially hypoxic conditions can complicate chemo- and radiotherapy treatment. Chemotherapeutics specifically targeting hypoxic tumor regions are currently under investigation as tumor-selective treatment strategies. The mode of action of BA has only been partially examined: the proapoptotic effects of BA were realized through activation of the mitochondrial apoptosis pathway via activation of caspases, induction of reactive oxygen species (ROS) and DNA damage [6]. In a comparison with transcriptome data in 60 National Cancer Institute (NCI) cell lines it was suggested that BA acts via various mechanisms such as cell cycle regulation, signal transduction, trans- and post-transcriptional regulation, protein biosynthesis, ubiquitination and proteasomal degradation [9]. Additionally, BA affects some transcription factors that promote tumor growth, metastasis, angiogenesis and drug resistance. For instance, BA inhibited the oncogenic STAT3 and NF-κB pathways and promoted p53 expression, which is responsible for reduced proliferation, enhanced apoptosis and attenuated angiogenesis in prostate cancer and oral squamous carcinoma cell lines [10,11,12]. So far, the importance of p53 for BA-induced apoptosis is not clear, because some studies reported that the efficiency of BA is independent of p53 [13]. However, the p53 pathway is one of the most important factors in cancer development and progression, as it enables the arrest of cancer cell growth, induction of apoptosis and activation of DNA repair.

Sestrin-2 (SESN-2) is a conserved antioxidant protein that is induced due to cellular stresses, such as oxidative stress (ROS), DNA damage, hypoxia or ER stress. Up-regulated *SESN2* expression is a mechanism to compensate cell damage and provide cytoprotective effects through inhibition of ROS and activation of autophagy [14]. *SESN2* expression is activated by many transcription factors which are activated by oxidative stress, such as nuclear factor p53, (erythroid-derived 2-) like 2 (Nrf2), activator protein 1 (AP-1) and hypoxia-inducible factor 1 (Hif-1a) [15]. SESN2 enhances the activation of AMP-activated protein kinase (AMPK), which leads to the inhibition of mTOR resulting in activation of autophagy [16]. In colorectal, hepatocellular and lung cancer, SESN2 is characterized as a tumor suppressor because of its decreased expression, which is associated with advanced tumor stage, metastasis and poor survival of cancer patients [17,18,19]. Furthermore, in colorectal cancer lentiviral overexpression of *SESN2* inhibited proliferation, enhanced apoptosis and reduced clonogenic survival in vitro and suppressed tumor growth in vivo [20]. In other cancers such as prostate and bladder cancer, increased SESN2 expression is also associated with attenuated proliferation, increased apoptosis and autophagy as well as anchorage-independent growth [21]. However, some other studies postulated higher SESN2 expression levels in tumor tissue compared to adjacent non-cancerous tissue were partially associated with poor outcomes for patients with lung, endometrial and hepatocellular cancer [22,23,24]. Consequently, the role of SESN2 in cancer prognosis and progression is not clear, particularly for chemo- and radioresistance. Preliminary studies indicate the effects of SESN2 on the radiosensitivity of prostate and breast cancer cells [25,26]. In osteosarcoma and lung cancer, the inhibition of SESN2 expression enhanced the cytotoxic effects of chemotherapeutic drugs [22,27].

In this study, expression analyses of BA-treated breast cancer cells under normoxic and hypoxic conditions identified *SESN2* as one of the most strongly up-regulated genes. Therefore, we investigated the effects of *SESN2* knockdown in combination with BA treatment on breast cancer proliferation, clonogenic survival, autophagy, ROS, DNA damage and radiosensitivity. 

## 2. Materials and Methods

### 2.1. Cell Culture Conditions and Treatment of Cells

Human breast cancer cell lines (MDA-MB-231, MCF-7, HS578T, Cal51, T47D) were cultured with RPMI (Thermo Fisher Scientific, Waltham, MA, USA) containing 10% fetal bovine serum (Capricorn Scientific, Ebsdorfergrund, Germany), 1% sodium pyruvate (Gibco, Thermo Fisher Scientific) and 2% penicillin/streptomycin (Sigma-Aldrich, St. Louis, MO, USA) at 37 °C and 5% CO_2_. The cells were grown in logarithmic growth phase and tested for mycoplasma contamination regularly. MCF-7 cells were purchased from CLS (Cell Lines Service GmbH, Eppelheim, Germany) and MDA-MB-231, MCF-7, HS578T, T47D and Cal51 were obtained from Prof. Dittmer from Department of Gynecology (Martin Luther University Halle-Wittenberg). Cell line authentication was achieved by genetic profiling using polymorphic short tandem repeat (STR) loci. Hypoxic culturing was realized by incubation of the cell culture flask in GasPack^TM^ systems (BD, Franklin Lakes, NJ, USA). Irradiation of breast cancer cells was performed with Oncor Impression IMRT (Siemens, Munich, Germany) at a dose rate of 2 Gy/min. 

Betulinic acid (BA) was dissolved in DMSO to achieve a concentration of 20 mM stock solution. For experimental setup, we used working concentrations of 10 µM and 20 µM and cells were treated for 24 h or 48 h, respectively.

### 2.2. Microarray Analysis

Microarray analysis was conducted on at least three samples treated with 10 µM BA for 48 h under normoxic (21% O_2_) and hypoxic (0.1% O_2_) conditions. Treatment was performed in mtp53 MDA-MB-231 and wtp53 MCF-7 breast cancer cell lines. 

RNA integrity and concentration were examined on an Agilent 2100 Bioanalyzer (Agilent Technologies, Palo Alto, CA, USA) using the RNA 6.000 LabChip Kit (Agilent Technologies) according to the manufacturer’s instructions. Briefly, after probe synthesis using the TargetAmp™- Nano Labeling Kit for Illumina Expression BeadChip (Epicenter Biotechnologies, Madison, WI, USA), cRNA was hybridized to Illumina HT-12 v4 Expression BeadChips (Illumina, San Diego, CA, USA) and scanned on the Illumina HiScan instrument according to the manufacturer’s specifications. PD Dr. Krohn performed Illumina BeadChip analysis at the microarray core facility of the Interdisciplinary Center for Clinical Research (IZKF) Leipzig (Faculty of Medicine, University of Leipzig).

The Illumina BeadChip microarray analysis was performed as previously described in Weinholdt et al. [28]. The *read.ilmn*-function of the limma package [29] was used to read the 48,210 microarray probes into R. Afterwards, limma’s *neqc*-function was used to perform a background correction followed by quantile normalization, using negative control probes for background correction, and both negative and positive controls for normalization. The 23,768 array probes corresponding to 17,218 genes, which displayed a significant hybridization signal (Illumina signal detection statistic at *p* < 0.05) in all probes, were used for further analysis. The differential expression analysis was performed using the limma functions *lmfit()* and *eBayes()*, and genes were defined as significantly regulated if the false discovery rate (FDR) was below 0.05 and the log2-fold-change to DMSO control exceeded 0.5 in magnitude. The KEGG pathway analyses were performed using DAVID Bioinformatics (Resources 6.7) [30], and pathways were defined as significantly enriched if the FDR was below 0.05. The structure of the data based on the log-expression was examined using the principal component analysis (PCA), and the magnitude of the log2-fold-changes was shown as color in a heat map.

### 2.3. siRNA Transfection and BA Treatment

Breast cancer cells were transfected with Silencer^®^ Select siRNA against *SESN2* (Assay ID: s38097, Thermo Fisher Scientific) 24 h after plating. After the former titration, we applied 20 nM of SESN2 siRNA by use of INTERFERin^®^ transfection reagent (Polyplus transfection, Illkirch-Graffenstaden, France) following the manufacturer’s instructions and as described previously [31]. A siRNA with a scrambled sequence (src siRNA) was used as nonsense control at the same concentration as the targeting siRNA. 24 h after transfection, cells were incubated with 10 µM or 20 µM BA, respectively, for another 24 h or 48 h depending on the performed assay. 

### 2.4. Analysis of RNA Expression

Breast cancer cells were harvested for RNA expression analysis with trizol method by use of Direct-zol RNA Miniprep Kit (ZymoResearch, Irvine, CA, USA) following the manufacturer’s instructions. Concentration and purification of RNA was determined with Nanodrop (Thermo Fisher Scientific). cDNA synthesis was performed as described previously [32]. QRT-PCR was realized with a TaqMan primer system (Thermo Fisher Scientific, Table 1) for validation of microarray results in five breast cancer cell lines and dye-based qPCR with Luna Universal qPCR Master Mix (Cell Signaling Technology, Inc., Danvers, MA, USA) for validation of siRNA transfection experiments. Sequences of primers used for qRT-PCR were purchased from Sigma-Aldrich and shown in Table 2. *MMGT1* (membrane magnesium transporter 1) and *HPRT* (hypoxanthine-guanine phosphoribosyltransferase) were used as housekeeping genes. No template control was used as a negative control.

For quantification of all RT-PCR data, the delta delta Ct method (ΔΔCt) was used [33]. ΔCt was calculated as the difference in the Ct value of the gene of interest and the Ct value of the reference gene (*MMGT1* or *HPRT*). The average Ct value of the normoxia or hypoxia DMSO-treated cells was chosen as the calibrator sample. ΔΔCt was defined as the difference in ΔCt of the treated probe and ΔCt of the calibrator probe. 2^−ΔΔCt^ displays the fold change of the mRNA expression level of the treated sample to the averaged calibrator sample.

### 2.5. Analysis of Protein Expression

Western blot was performed as described previously [32]. Breast cancer cells were scraped off with cell lysis buffer (Cell Signaling) supplemented with protease inhibitor (Cell Signaling) and phosphatase inhibitor (Thermo Fisher Scientific). Afterwards cells were homogenized by ultrasound and Bradford method (Bio-Rad Laboratories, Inc., Hercules, CA, USA) was used for determination of protein concentration. Proteins were separated by SDS gel electrophoresis followed by transfer to polyvinylidene fluoride (PVDF) membranes (Merck Millipore, Burlington, MA, USA) using a tank blot system (Bio-Rad Laboratories). Membranes were blocked with 10% nonfat milk/TBST (150 mM NaCl, 50 mM Tris-HCl pH 7.5, 0.1% Tween-20) for 1 h at room temperature and incubated with primary antibodies against SESN2 (1:2000; Proteintech, Rosemont, IL, USA) and actin (1:5000; Sigma-Aldrich) overnight at 4 °C. We used HRP-conjugated secondary antibodies (goat anti-rabbit; 1:5000; Dako Deutschland GmbH, Hamburg, Germany; rabbit anti-mouse; 1:5000; Cell Signaling). An ECL detection system (Advansta Inc., San Jose, CA, USA) was used to visualize immune complexes with a ChemiDoc Imaging System (Bio-Rad). For multiple detection of different antibodies, membrane was stripped in stripping buffer (62.5 mM Tris-HCl pH 6.8, 2% SDS, 0.75%-mercaptoethanol) for 20 min at 50 °C using a hybridization oven, blocked again and reprobed with primary antibodies.

### 2.6. Cytotoxicity

Sulforhodamine B (SRB) assay was used to determine the influence of *SESN2* knockdown on the cytotoxicity of BA. Therefore, cells were seeded at a defined cell number, depending on cell line (MDA-MB-231, MCF-7 and HS578T) and oxygen conditions, in 96 well plates. After 24 h, cells were transfected with siRNA as described previously, and after an additional 24 h, growth medium was changed and cells were treated with different concentrations of BA (0–40 µM) for another 48 h. Afterwards, cells were fixed, washed and stained as described previously [32]. Extinction was measured at 540 nm and IC50 (half-maximal inhibitory concentration) values were calculated by dose response curve fitting using Origin 2019 (OriginLab Corp., Northampton, MA, USA). 

### 2.7. Clonogenic Survival and Radiosensitivity

Clonogenic survival assay was performed 24 h after siRNA transfection followed by 24 h BA treatment. For determination of radiosensitivity, cells were additionally irradiated under hypoxic conditions (0.1% O_2_) at different doses (0 Gy–14 Gy) depending on cell line. Procedure, survival fraction (SF), dose modifying factor (DMF10) and enhancement factor (EF) were conducted as described previously [32].For colony counting, GelCount system (Oxford Optronics, Abingdon, UK) was used. Cell survival curves were fitted to a linear quadratic model (-lnS = αD + βD^2^) using Origin 2019.

### 2.8. Analysis of Autophagy: Bafilomycin Clamp Assay

LC3B (microtubule associated protein 1 light chain 3 beta) is conjugated to phosphatidylethanolamine during phagophore formation and remains attached to the autophagosomal membrane until its fusion with the lysosome. In order to differentiate whether increased LC3B levels are due to enhanced autophagosome formation or an impaired lysosomal degradation, cells are treated with and without Bafilomycin (BafA1). BafA1 inhibits lysosomal acidification and thus prevents LC3B degradation [34]. Procedure for determination of autophagic flux (bafilomycin clamp assay) was modified to Sharifi et al. [35]. Breast cancer cells were seeded in a μ-Slide 8 well glass bottom coverslip with a density of 3 × 10^4^ cells per well (Ibidi, Gräfelfing, Germany). After siRNA transfection and BA treatment for 24 h in the absence or presence of 20 nM Bafilomycin A1 (Sigma-Aldrich), breast cancer cells were washed with PBS and fixed and permeabilized with ice cold methanol at 4 °C for 15 min and washed three times afterwards with PBS. Cells were then blocked with 1% BSA/PBS at room temperature for 1 h and subsequently incubated with LC3B antibody diluted in 1% BSA/PBS (1:200; #2775, Cell Signaling) at 4 °C overnight. After three washing steps with PBS, cells were incubated with secondary antibody Alexa Fluor^®^ 555 anti-rabbit (1:800, Invitrogen, Thermo Fisher Scientific) at room temperature for 1 h before DAPI staining (1 µg/mL, Carl Roth, Karlsruhe, Germany) for 5 min in the dark at room temperature. Coverslips were mounted with fluorescence mounting medium (Dako Deutschland GmbH). LC3B puncta were detected using a fluorescence microscope (Keyence BZX800). with 40x magnification and exposure times DAPI 0.5 s; TRITC: 3.5 s. The number of LC3B puncta and cell nuclei was quantified using the ‘analyze particles’ tool in ImageJ (version 1.53) in at least 100 cells. Autophagy flux is calculated as the difference between the number of detected LC3B puncta in Baf1A-coincubated cells and cells treated without BafA1.

### 2.9. Analysis of ROS

Level of intracellular ROS was measured by the incubation of cells with specific CM-H2DCFDA dye (Thermo Fisher Scientific) [36]. After siRNA transfection and BA treatment, breast cancer cells (MDA-MB-231, MCF-7, HS578T) were stained with 0.5 µM CM-H2DCFDA (in PBS supplemented with CaCl_2_ and MgCl_2_, Sigma-Aldrich) for 30 min at 37 °C. Detached cells were analyzed for fluorescent signal with LSRFortessa™ flow cytometer (BD Biosciences, Heidelberg, Germany).

### 2.10. Quantification of γH2AX Foci Formation

Formation of yH2AX foci directly correlates with the number of DNA double-strand breaks (DSBs). DSB can be caused, for example, by chemotherapeutics and most of them were frequently repaired. Foci still existing after 24 h are so-called residual γH2AX foci that mark unrepaired DSBs, which play an important role for the radiosensitivity of cells [37]. Breast cancer cells were seeded in a μ-Slide 8 well glass bottom coverslip with a density of 3 × 10^4^ cells per well. After siRNA transfection and BA treatment for 24 h, cells were irradiated with 0 Gy and 4 Gy followed by further incubation under hypoxia for 24 h. Residual γH2AX foci were analyzed by immunofluorescence as described previously [36].

### 2.11. Statistical Analysis

All data represent the mean value and standard deviation (+SD) of at least three independent experiments. The significance of differences was assessed using an unpaired two-tailed Student’s *t*-test. A p value was considered to indicate a significant difference in reference to the indicated population of control cells. Significant p values are highlighted with asterisks (* *p* < 0.05, ** *p* < 0.01, *** *p* < 0.001).

## 3. Results

### 3.1. Microarray Analysis

**Figure 1:**
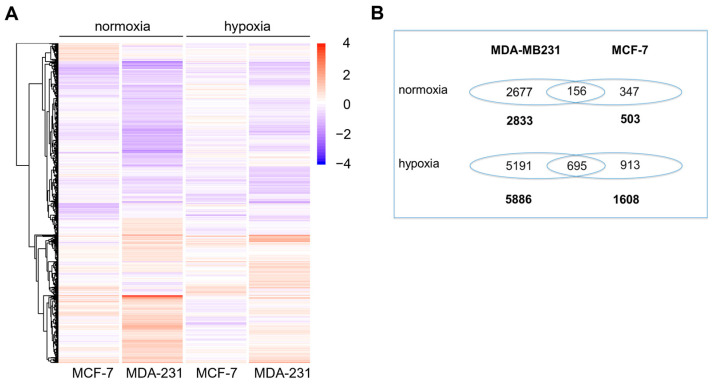
Heat map and Venn diagram of 10 µM BA-treated cells. Transcriptomic profiles of breast cancer cell lines under normoxia and hypoxia used cDNA microarray are presented. MDA-MB-231 and MCF-7 cells were incubated with 10 µM BA under normoxia (21% O_2_) and hypoxia (0.1% O_2_) for 48 h. (**A**) Heat map displays genes up-regulated relative to DMSO treated control cells in red and down-regulated genes in blue. (**B**) Differential gene expression was calculated as log2-fold-change > 0.5 or <−0.5 of DMSO control cells in at least three independent experiments (*p* < 0.05).

To identify pathways and genes that are probably involved in the mode of action of BA, two human breast cancer cell lines MDA-MB-231 (mtp53) and MCF-7 (wtp53) were treated with BA for 48 h under normoxic and hypoxic conditions. A heat map and Venn diagram showed differently expressed genes (DEGs) after BA treatment under normoxic or hypoxic conditions in both breast cell lines (Figure 1). Under normoxic conditions, 2833 and 503 DEGs were identified in BA-treated MDA-MB-231 and MCF 7 cells based on log2-fold-change >0.5 or <−0.5 (*p* < 0.05) (Figure 1B), respectively. BA treatment deregulated 2.1-fold and 3.2-fold more genes in MDA-MB-231 (mtp53) and MCF 7 (wtp53) cells under hypoxic conditions compared to normoxia. A total of 156 (normoxia) or 695 (hypoxia) DEGs were detected after BA treatment in both cell lines (Figure 1B). Principal component analysis (PCA) is shown in Supplementary Figure S1.

MCF-7 cells showed stronger response to hypoxic conditions than MDA-MB-231 cells. KEGG pathway analysis revealed ten significantly dysregulated pathways in MCF-7 cells under hypoxia compared to normoxia but only four in MDA-MB-231 cells (Table 3). In contrast, MDA-MB-231 cells showed a higher impact on pathways after BA incubation than MCF-7 cells. In MDA-MB-231 cells, six and twelve significant dysregulated signaling pathways were identified after BA treatment under normoxia and hypoxia, respectively. In contrast, in MCF-7 cells only three KEGG signaling pathways were BA-induced under normoxia and none under hypoxia.

In detail, cell cycle, DNA replication and base excision repair are hypoxia-regulated KEGG pathways in both breast cancer cell lines. In addition, the cell cycle and DNA replication pathways are also induced after BA treatment in both breast cancer cell lines (Table 3). The p53 and pyrimidine KEGG pathway are activated under hypoxic conditions in wtp53 cell line MCF-7. In the mtp53 cell line MDA-MB-231, these two pathways are just stimulated by additional BA treatment under hypoxia (Table 3). Further, Table 3 shows additional pathways specifically regulated after BA treatment under normoxia or hypoxia in either MDA-MB-231 or MCF-7 cells.

**Figure 2:**
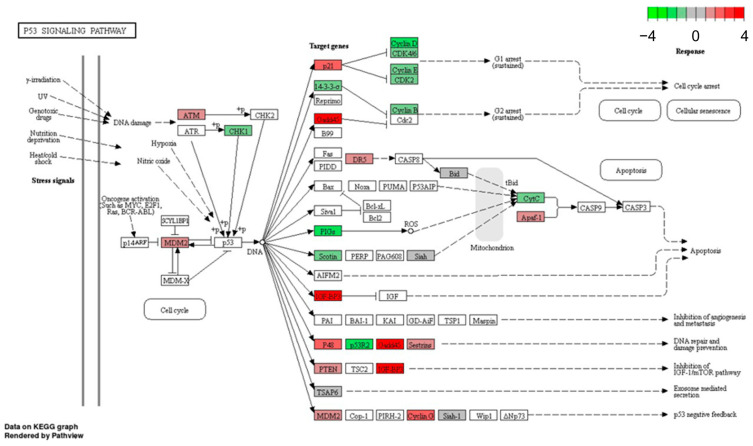
Hypoxia induced p53 KEGG pathway in MCF-7 cells (wtp53) (Benjamini-Hochberg adjusted *p* < 0.05). Apaf-1, apoptotic peptidase activating factor 1; ATM, ataxia telangiectasia mutated; CDK2/4/6, cyclin-dependent kinase 2/4/6; CHK1, cell cycle checkpoint kinase; CytC, cytochrome C; DR5, death receptor 5; GADD45, growth arrest and DNA damage-inducible protein 45; IGFBP3, insulin-like growth factor-binding protein 3; MDM2, mouse double minute 2 homolog; PIGs, p53-induced gene; PTEN, phosphatase and tensin homolog; p21 (=CDKN1A), cyclin-dependent kinase inhibitor 1A; p48 (=PTF1A), pancreas associated transcription factor 1a; p53R2 (=RRM2B), ribonucleotide reductase regulatory TP53 inducible subunit M2B); SESN2, sestrin-2; Siah-1, seven in absentia homolog 1; TSAP6 (=STEAP3), tumor suppressor-activated pathway protein 6; 14-3-3σ, Stratifin.

**Figure 3:**
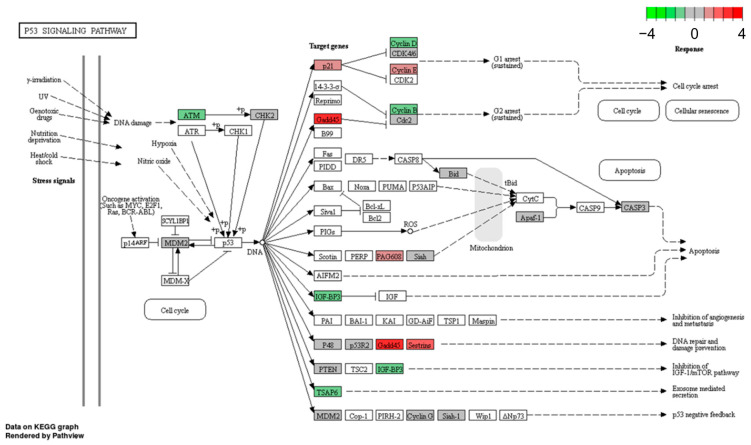
BA-induced p53 KEGG pathway in MDA-MB-231 cells (mtp53) under hypoxia (Benjamini-Hochberg adjusted *p* = 0.01). Apaf-1, apoptotic peptidase activating factor 1; ATM, ataxia telangiectasia mutated; Bid, BH3 interacting domain death agonist; CASP3, Caspase 3; CDK4/6, cyclin-dependent kinase 4/6; Cdc2 (=CDK1), cell division cycle 2; CHK2, cell cycle checkpoint kinase; GADD45, growth arrest and DNA damage-inducible protein 45; IGFBP3, insulin-like growth factor-binding protein 3; MDM2, mouse double minute 2 homolog; PAG608 (ZMAT3), p53-activated gene 608 protein; PTEN, phosphatase and tensin homolog; p21 (=CDKN1A), cyclin-dependent kinase inhibitor 1A; p48 (=PTF1A), pancreas associated transcription factor 1a; p53R2 (=RRM2B), ribonucleotide reductase regulatory TP53 inducible subunit M2B); SESN2, sestrin-2; Siah-1, seven in absentia homolog 1; TSAP6 (=STEAP3), tumor suppressor-activated pathway protein 6.

We selected the p53 signaling pathway for detailed investigations. While the p53 pathway is already activated by hypoxia (without BA) in the wtp53 cell line MCF-7 (Table 3), in mtp53 cell line MDA-MB-231 cells this pathway is just stimulated under hypoxia by additional BA treatment (Table 3). Detailed KEGG pathway analysis showed that DEGs (up- and down-regulated) of the p53 signaling pathway that are dysregulated in MCF-7 cells under hypoxia (Figure 2) are similar to those regulated by BA treatment under hypoxia in MDA-MB-231 cells (Figure 3). 

### 3.2. Validation of Microarray Data

Fourteen genes of the p53 signaling pathway that were significantly up- or down-regulated by hypoxia and/or BA treatment in MCF-7 and MDA-MB-231 breast cancer cells (Figure 2 and Figure 3) were selected for validation of microarray analysis by qPCR in five breast cancer cell lines. 

Enhanced expression of *GADD45A/B/G, SESN2, CDKN1A, CCNE1, PMAIP1, ZMAT3* and decreased expression of *ATM, IGFBP3, STEAP3, CCND1, CCND2, CDK6* were induced by BA treatment in MDA-MB-231 (mtp53) and MCF-7 (wtp53) cells by normoxia and/or hypoxia (Table 4). Comparison of gene expression levels (log2-fold-change) measured by qRT-PCR and microarray indicated a strong correlation (Supplementary Figure S2).

Similar expression levels after BA treatment were detected in three additional breast cancer cell lines HS578T (mtp53), T47D (mtp53) and Cal51 (wtp53) (Table 4). It is striking that the mtp53 cell lines MDA-MB-231 and T47D showed the most differentially regulated genes, whereas mtp53 cell line HS578T and wtp53 cell lines MCF-7 and Cal51 showed less regulated genes (Table 4). Genes of *GADD45* family and *SESN2* were up-regulated in all investigated breast cancer cell lines after BA treatment. In addition, the effects of hypoxia alone on the selected genes in five breast cancer cell lines are presented in Supplementary Table S1.

**Figure 4:**
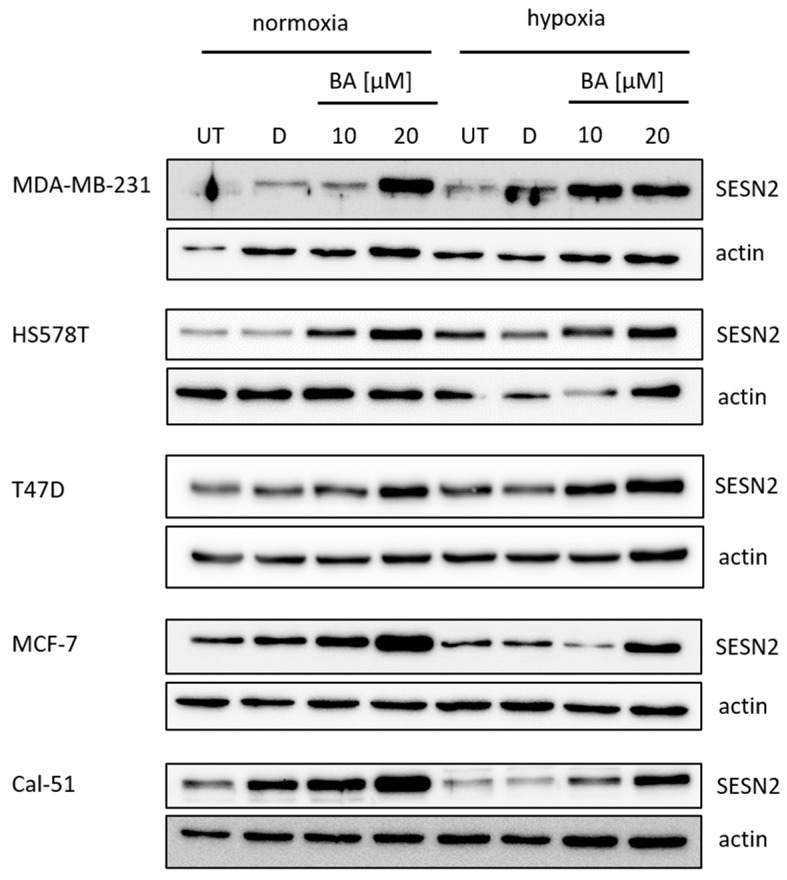
Western blot of SESN2 protein expression. Five breast cancer cell lines with different p53 status were analyzed for SESN2 (54 kDa) protein level after treatment with 10 µM and 20 µM BA under normoxic (21% O_2_) and hypoxic (0.1% O_2_) conditions for 48 h. A representative blot of at least three independent experiments is shown for each cell line. Actin (42 kDa) was used as a loading control. UT—untreated; D—DMSO.

The *SESN2* gene is one of the strongest up-regulated genes after BA treatment in all five investigated breast cancer cell lines (Table 4). In addition, protein expression level was examined after BA treatment. Western blot analysis revealed also an increase in SESN2 protein level after BA treatment in all five investigated breast cancer cell lines under normoxic and hypoxic conditions (Figure 4). 

### 3.3. Knockdown of BA-Induced SESN2 Expression

**Figure 5:**
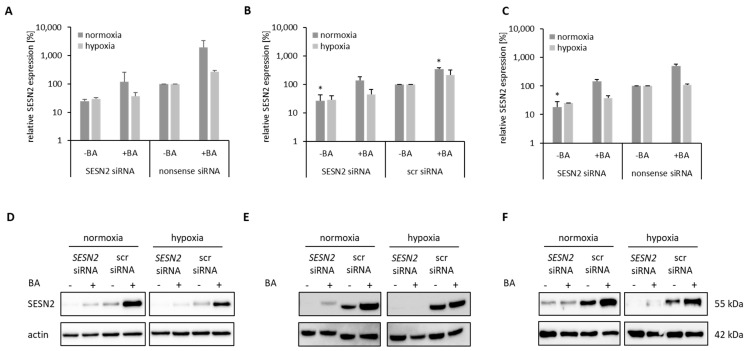
Knockdown of SESN2 expression in human breast cancer cells. Breast cancer cells were transfected with siRNA against *SESN2* and additionally treated with 20 µM BA for another 24 h under normoxic and hypoxic conditions. A-C. qRT-PCR: Effective *SESN2* knockdown is shown without and after BA treatment in MDA-MB-231 (**A**), HS578T (**B**) and MCF-7 (**C**) cell line. Data represent mean values (+SD) of at least three independent experiments. Significant p values are highlighted with asterisks (* *p* ≤ 0.05). D-F. Western blot of MDA-MB-231 (**D**), HS578T (**E**) and MCF-7 (**F**) cells: BA treatment induced SESN2 (55 kDa) protein level, which is prevented by *SESN2* knockdown. A representative blot is shown and Actin (42 kDa) is used as a loading control. src siRNA – scrambled siRNA.

Breast cancer cell lines MDA-MB-231, HS578T and MCF-7 were transfected with 20 nM siRNA against *SESN2* under normoxic and hypoxic conditions. *SESN2* mRNA expression level was reduced to 20–30% independently of oxygen conditions (Figure 5A–C). Although BA treatment increased *SESN2* mRNA expression level by at least a factor of three, prior knockdown of *SESN2* prevented BA-mediated induction of *SESN2*. Concerning Western blot analysis, a reduced SESN2 level was also detected after siRNA transfection, and siRNA transfection prior to BA treatment prevented BA-mediated SESN2 induction in all three breast cancer cell lines (Figure 5D–F).

### 3.4. Cytotoxicity of SESN2-Silenced Breast Cancer Cells

We performed SRB assay and clonogenic survival assay to investigate the effect of *SESN2* knockdown on the cytotoxicity of BA treatment in the three breast cancer cell lines, MDA-MB-231, MCF-7 and HS578T, under normoxic and hypoxic conditions. As described previously, we found elevated cytotoxicity of BA under hypoxic conditions compared to normoxia [7]. However, we could not detect any additional effect of *SESN2* knockdown on the cytotoxicity or clonogenic survival of BA treatment (data not shown).

### 3.5. Effect of SESN2 Silencing on Autophagy Induction in BA-Treated Breast Cancer Cells

**Figure 6:**
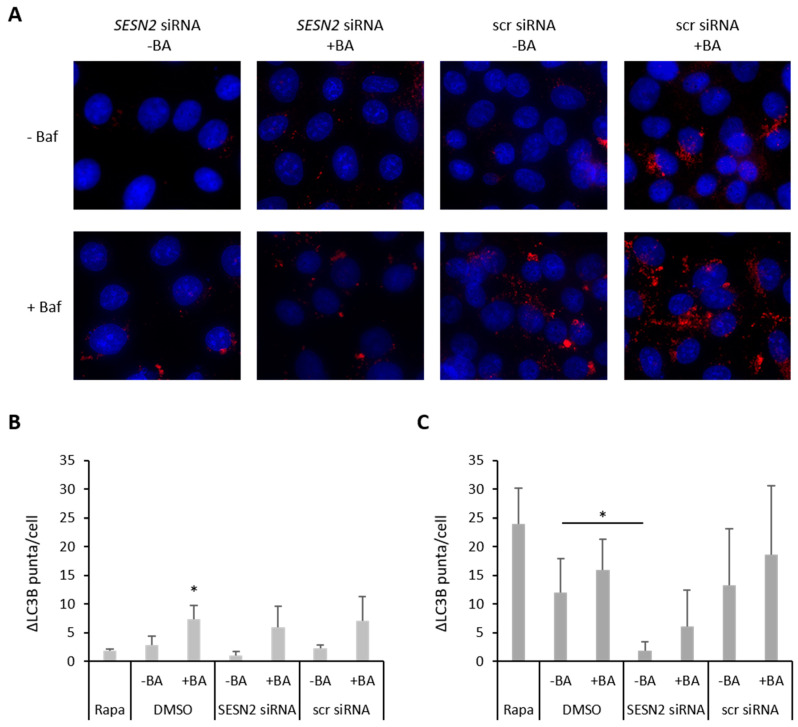
Bafilomycin-Clamp-Assay. MDA-MB-231 cells were transfected with siRNA against *SESN2* and treated with BA under hypoxic conditions (0.1% O_2_) with and without BafA1. LC3B puncta per cell were counted out of at least 100 cells. (**A**) Representative immunofluorescence staining of LC3B (red) and cell nuclei (blue: DAPI). (**B**) LC3B puncta per cell after siRNA and BA treatment without BAf1A. (**C**) Autophagic flux (ΔLC3B puncta) of MDA-MB-231 cells under hypoxic conditions. Data represent mean values (+SD) of three independent experiments. All data were referred to scr siRNA without BA treatment. Significant p values are highlighted with asterisks (* *p* ≤ 0.05).

One of the most important proteins for autophagy is LC3B, which can be detected by Western blot, immunhistochemistry or via immunofluorescence. We chose immunofluorescence-based LC3B-Bafilomycin-clamp-assay (Figure 6A). *SESN2*-silenced and/or BA-treated cells were incubated with and without V-ATPase inhibitor Baf1A to investigate LC3B level (Figure 6B) and autophagic flux (Figure 6C) by counting LC3B puncta per cell. BA treatment caused a significant increase in LC3B level (3.3-fold, *p* = 0.03). *SESN2* knockdown caused a slight decrease in LC3B level (0.5-fold) and an attenuated BA-mediated increase in LC3B level (Figure 6B). However, an increased LC3B level might be caused by enhanced autophagosome formation (increase in autophagic activity) or an impaired lysosomal degradation (decrease in autophagic activity). Additional treatment of breast cancer cells with Baf1A, an inhibitor of autophagosome-lysosome fusion, enabled us to determine autophagic flux (Figure 6C): Treatment with autophagy inducer rapamycin resulted in a 2.2-fold increase in autophagic flux. BA treatment caused a 1.5-fold increase in autophagic flux, whereas breast cancer cells with silenced *SESN2* expression showed only a 0.2-fold (*p* = 0.1) autophagic flux compared to scr siRNA treated cells. Furthermore, *SESN2* silencing prevented a BA-induced increase in autophagic flux (Figure 6C). 

### 3.6. SESN2 Silencing Enhances ROS Production of BA-Treated Breast Cancer Cells

**Figure 7:**
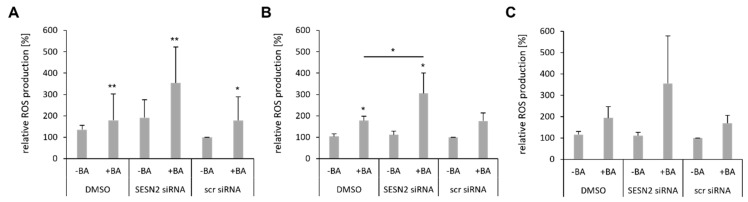
SESN2 silencing enhances ROS production of BA-treated breast cancer cells. Breast cancer cell lines MDA-MB-231 (**A**), HS578T (**B**) and MCF-7 (**C**) were transfected with siRNA against SESN2 and treated with BA under hypoxic conditions (0.1% O_2_). Intracellular ROS level was measured with CM-H2DCFDA staining. Data represent mean values (+SD) of at least three independent experiments. All data were referred to scr siRNA without BA treatment (=100%). Significant p values are highlighted with asterisks (* *p* ≤ 0.05; ** *p* ≤ 0.01).

Cultivation of breast cancer cells under hypoxic conditions (0.1% O_2_) caused an increase in cellular ROS (data not shown). Additionally, BA induced a twofold increase in ROS in MDA-MB-231 (** *p* = 0.007), HS578T (** *p* = 0.002) and MCF-7 (*p* = 0.09) breast cancer cells under hypoxia (Figure 7). It is known that cellular stress such as hypoxia or oxidative stress (ROS) induce SESN2 expression to mediate cytoprotection [15]. Only in MDA-MB-231 cells, the silencing of *SESN2* causes a slight increase (1.9-fold) in intracellular ROS under hypoxic conditions (Figure 7A). We hypothesize that preventing BA-mediated SESN2 induction via siRNA transfection induces ROS production additionally. In all three cell lines, *SESN2* silencing caused a 3-3.5-fold increase in intracellular ROS (Figure 7). 

### 3.7. SESN2 Silencing Enhances DNA Damage of BA-Treated Breast Cancer Cells

**Figure 8:**
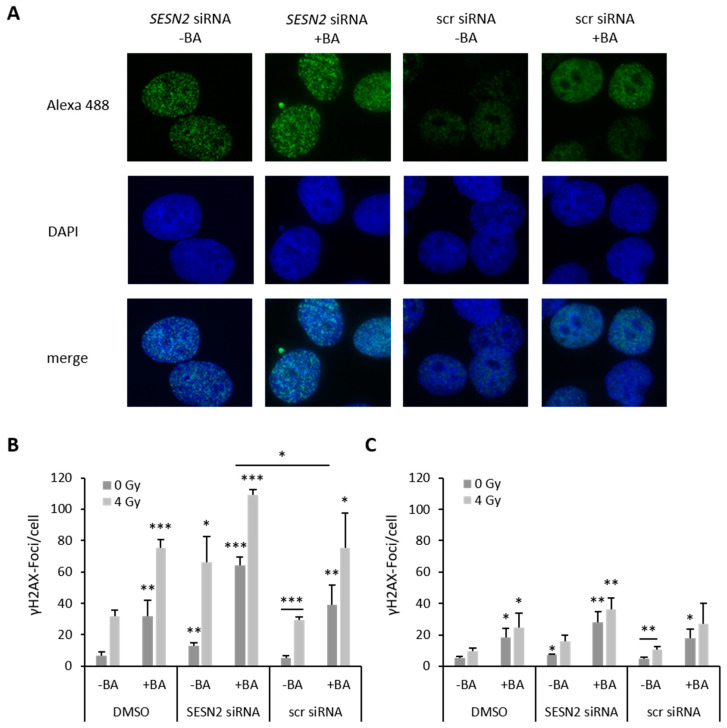
SESN2 silencing enhances DNA damage of BA-treated breast cancer cells. (**A**) Representative immunofluorescence staining of γH2AX foci (green) and cell nuclei (DAPI: blue) of non-irradiated MDA-MB-231 cells. Breast cancer cell lines MDA-MB-231 (**B**) and HS578T (**C**) were transfected with siRNA against *SESN2,* treated with BA and irradiated with 4 Gy under hypoxic conditions (0.1% O_2_). γH2AX foci per cell were counted out of 100 cells. Data represent mean values (+SD) of at least three independent experiments. All data were referred to scr siRNA without BA treatment of non-irradiated (0 Gy) or irradiated cells (4 Gy). Significant p values are highlighted with asterisks (* *p* ≤ 0.05; ** *p* ≤ 0.01; *** *p* = 0.001).

BA treatment caused an 8.7-fold (*p* = 0.009) and 3.7-fold (*p* = 0.04) increase in γH2AX foci in MDA-MB-231 (Figure 8B) and HS578T cells (Figure 8C). Inhibiting *SESN2* expression also induced formation of γH2AX foci in MDA-MB-231 (2.7-fold, *p* = 0.006) and in HS578T cells (1.5-fold, *p* = 0.02). A combination of BA treatment and *SESN2* silencing obtained the strongest induction of γH2AX foci formation in MDA-MB-231 (13.6-fold, *p* ≤ 0.001) and HS789T cells (5.8-fold, *p* = 0.003). Additional irradiation of breast cancer cells with 4 Gy induced formation of γH2Ax foci significantly (Figure 8) and reinforced the effects of BA treatment and/or *SESN2* inhibition. 

### 3.8. Radiosensitization of BA-Treated Breast Cancer Cells after SESN2 Silencing

**Figure 9:**
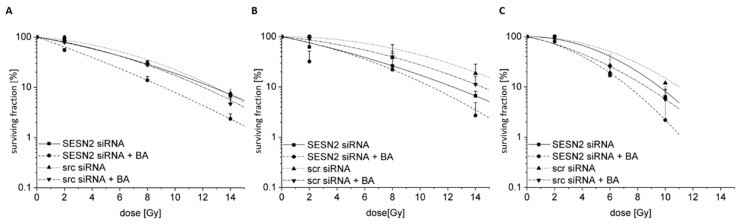
Radiosensitivity of breast cancer cells. Breast cancer cells ((**A**) MDA-MB-231, (**B**) HS578T, (**C**) MCF-7) were transfected with siRNA against *SESN2* and treated with 20 µM BA under hypoxic conditions (0.1% O_2_). 24 h after BA treatment cells were irradiated under hypoxic conditions with different doses depending on cell line. Afterwards, 150 to 10,000 single cells were plated depending on cell line and radiation dose. After approximately 12 days, colonies were stained and counted. Data represent mean values (+SD) of at least three independent experiments.

BA treatment of human breast cancer cells had almost no effect on radiosensitization (Figure 9) [7]. *SESN2* knockdown resulted in a slight radiosensitization of HS578T (DMF10 = 1.67 ± 0.47, *p* = 0.1) and MCF-7 cells (DMF10 = 1.18 ± 0.10, *p* = 0.3) under hypoxia. 

However, the combination of BA treatment and *SESN2* inhibition caused a significant radiosensitization of MDA-MB-231 (DMF10 = 1.40 ± 0.18, *p* ≤ 0.01), HS578T (DMF10 = 3.15 ± 1.80, *p* = 0.01) and MCF-7 cells (DMF10 = 1.20 ± 0.004, *p* = 0.3) under hypoxia. The radiosensitizing effect of *SESN2* knockdown in combination with BA treatment is even stronger at higher doses (10 Gy or 14 Gy). Under oxidative stress, such as hypoxia, SESN2 has cytoprotective effects. Silencing *SESN2* means a loss of cytoprotective effect and results in elevated radiosensitivity. 

## 4. Discussion

BA is a well-described natural compound with numerous anti-tumor effects. A comparison of transcriptional profiles with the cytotoxicity of BA in 60 NCI cell lines suggests the activation of different mechanisms [9]. In several studies, the pro-apoptotic, anti-proliferative, anti-migratory, anti-angiogenetic and radiosensitive effects of BA were described for different tumor entities (reviewed in [5,6]). The mode of action of BA includes processes such as induction of oxidative stress, cell cycle dysregulation, DNA damage, autophagy and apoptotic cell death and disruption of mitochondrial potential. BA treatment leads to the production of ROS, which results in the reduction of mitochondrial potential and pro-apoptotic proteins, such as Caspase-3 and Caspase-9, become up-regulated and induce apoptosis of tumor cells. Transcription factors, such as STAT, NF-κB and SP1, which play important roles in metastasis, proliferation, apoptosis, angiogenesis and drug resistance were also regulated by BA [5]. Besides the high selectivity of BA against tumor cells, an enhanced cytotoxicity under hypoxic conditions is described in human glioma and breast cancer cells [7,8]. Initial studies proved the inhibition of the accumulation of HIF-1α, one of the most important hypoxia-regulated proteins, by BA under hypoxic conditions in prostate, glioma, endometrial and cervical cancer [8,10,38,39]. The high cytotoxicity of BA in hypoxic tumor cells is a quite beneficial property, especially for cancer treatment, but the detailed mechanism is not known. Therefore, we performed microarray studies of BA-treated human breast cancer cells under normoxic and hypoxic conditions. Our KEGG pathway database revealed the p53 signaling pathway as one important pathway that is dysregulated after BA treatment under hypoxic conditions (Table 3 ). Nevertheless, a p53-independent mechanism for induction of apoptosis by BA has been postulated, because cytotoxic effects were observed in mutant and wildtype p53 cell lines (reviewed in [6]). In colorectal cancer cell lines with different p53 status, BA reduced p53 protein level in a dose-dependent manner, while in oral squamous cell carcinoma, p53 protein level was enhanced [11,40]. 

Expression analysis of 14 genes, which have been identified as dysregulated by BA treatment in microarray analysis before, revealed a strong up-regulation of the *GADD45* gene family (-A, -B, -G) and *SESN2* in all investigated human breast cancer cell lines with different p53 status. Additionally, our qRT-PCR analysis identified *SESN2* as one of the most strongly up-regulated genes after BA treatment (Table 4). A transcriptome study of breast cancer cells treated with oleanolic acid, which is also a triterpenoid and shows high structural similarities to BA, also identified *SESN2* as one of the most up-regulated genes [41]. Independent of p53 status, in wtp53 cell lines MCF-7 and Cal51, as well as in mtp53 cell lines MDA-MB-231, HS578T and T47D, *SESN2* expression was increased after BA treatment under normoxic and hypoxic conditions (Table 4 and Figure 4).

Although *SESN2* is induced downstream of wtp53, it has been shown that *SESN2* induction by ER stress, hypoxia, or chemotherapeutics might occur in a wtp53-independent manner [42,43,44]. Chemotherapeutics or radiation generate cellular stress, such as ROS, DNA damage or ER stress, which promotes *SESN2* expression [14,45]. However, tumor cells develop various mechanisms to overcome cellular stress, for example by up-regulating *SESN2* expression, which causes resistance to chemotherapeutics or radiation. We showed that BA induced ROS and DNA damage in breast cancer cells (Figure 7 and Figure 8). To investigate the importance of BA-induced *SESN2* expression, we reduced *SESN2* levels via siRNA and analyzed the therapeutic effects of BA in breast cancer cell lines. Our results indicate that SESN2 induction by BA has cytoprotective effects. The combined treatment of MDA-MB-231, HS578T, and MCF-7 cells with *SESN2* siRNA and BA resulted in enhanced DNA damage and ROS production as well as inhibited autophagy compared to single BA treatment. The cytoprotective function of SESN2 has already been shown in various studies. In prostate cancer, cell and fibroblast’s energetic stress-induced apoptosis was elevated after *SESN2* knockdown and restored after *SESN2* rescue [46]. Additionally, *SESN2* knockdown attenuated the proliferation and migration of lung cancer cells as well as enhancing the cytotoxic effects of ROS-inducing chemotherapeutic drugs (doxorubicin and cisplatin) [22]. Similar effects were observed after sorafenib or vemurafenib therapy in hepatocellular cancer cells or melanoma cells, respectively [24]. In accordance with our study, in osteosarcoma, the inhibition of chemotherapy-increased *SESN2* expression prevented chemoresistance through the inhibition of autophagy and induction of apoptosis [27,47]. However, there are other investigations that indicate the inhibitory effects of SESN2 in cancer. On the one hand, *SESN2* overexpression caused the inhibition of proliferation and induction of apoptosis in colorectal cancer cells [20], and on the other, the silencing of *SESN2* promoted migration, proliferation and attenuated apoptosis in endometrial cancer [23]. Additionally, drugs like 5-Fluorouracil or quercetin induced *SESN2* expression as well, but a *SESN2* knockdown attenuated drug-mediated effects in colon cancer cells [44,48]. These opposing studies show the dual function of SESN2 as an oncogene and tumor suppressor. SESN2 induction after BA treatment shall be assumed to be a side-effect of BA treatment due to stressful conditions that were generated by BA. However, SESN2 as a direct target gene of BA cannot be excluded and should be part of further investigations.

The relevance of SESN2 for tumor response to chemo- or radiotherapy is not clear. Some studies proposed that *SESN2* overexpression resulted in the enhanced radiosensitivity of human breast and prostate cancer cells [25,26]. Nevertheless, we found an enhanced radiosensitization of BA-treated hypoxic breast cancer cells when the expression of stress-induced SESN2 is depleted (Figure 9). This observation emphasizes the cytoprotective function of SESN2 after BA treatment. In glioblastoma and NSCLC cells, *SESN2* expression was elevated after irradiation, and *SESN2* knockdown sensitized cancer cells to irradiation as well [49,50]. Lin et al. identified that irradiation-induced miR-182-5p represses *SESN2* expression, which results in the enhanced ROS production and radiosensitivity of head and neck cancer cells. Furthermore, patients that received radiotherapy with high miR-182-5p and low *SESN2* expression had the most favorable prognosis [51]. 

Autophagy can be stimulated by SESN2 via the mTOR pathway and could be a factor explaining the protective function of SESN2 against BA treatment. Autophagy inhibition by chemical drugs, such as chloroquine, inhibited growth and enhanced tumor cell apoptosis [14]. Furthermore, in osteosarcoma cells, chemotherapy-induced *SESN2* expression promoted autophagy, which diminished tumor cell apoptosis. In *SESN2* silenced osteosarcoma cells, they observed inhibited autophagy and increased apoptosis, as well as reduced tumor progression, in a mouse model [27]. Autophagy may be part of a tumor cell mechanism to prevent cellular stress and to reach chemoresistance [14]. In our study, BA treatment caused an increase in autophagic flux, and attenuated autophagy after additional *SESN2* silencing (Figure 6) was accompanied by an increased radiosensitivity of breast cancer cells. However, the relationship between autophagy and chemotherapeutics or radiation remains debatable. Autophagy inducer rapamycin is used as an mTOR inhibitor for cancer therapy to decrease proliferation and increase autophagy. In addition, various studies have shown that treatment with autophagy inducers leads not only to radioresistance but also to radiosensitization of cancer cells (reviewed in [52]). Further analyses are necessary to investigate the distinct role of autophagy for radiosensitivity in the context of SESN2 in human breast cancer cells. In addition, due to the higher tolerance of normal cells to BA treatment, further investigations into the importance of SESN2 in normal tissue appear of interest. The highlight of this study is the identification and characterization of an important target gene, *SESN2*, to enhance the radiobiological effects (anti-tumor properties) of BA on breast cancer cells.

## 5. Conclusions

BA-induced *SESN2* expression decreased the anti-tumor effects of BA treatment. However, combined *SESN2* knockdown and BA treatment sensitized human breast cancer cells to radiation, enhanced apoptosis, DNA damage and ROS production and inhibited autophagy. SESN2 plays an important role in the effectiveness of BA treatment. The suppression of the antioxidant protein SESN2 influences the efficiency of radiotherapy in the presence of BA. Therefore, the combined therapy consisting of SESN2 knockdown or inhibition and BA treatment requires further study as a treatment strategy for breast cancer. 

## Figures and Tables

**Table 1:** TaqMan primer.

Gene	Gene ID	Assay ID	Product Length
*ATM*	472	Hs00175892_m1	89 bp
*CCND1*	595	Hs00765553_m1	57 bp
*CCND2*	894	Hs00153380_m1	69 bp
*CCNE1*	898	Hs01026536_m1	64 bp
*CDK6*	1021	Hs01026371_m1	64 bp
*CDKN1A*	1026	Hs00355782_m1	66 bp
*GADD45A*	1647	Hs00169255_m1	123 bp
*GADD45B*	4616	Hs00169587_m1	74 bp
*GADD45G*	10912	Hs00198672_m1	62 bp
*IGFBP3*	3486	Hs00426287_m1	66 bp
*MMGT1*	93380	Hs00298045_m1	72 bp
*PMAIP1*	5366	Hs00560402_m1	90 bp
*SESN2*	83667	Hs00230241_m1	65 bp
*STEAP3*	55240	Hs00217292_m1	61 bp
*ZMAT3*	64393	Hs00536976_m1	62 bp

ATM, ataxia telangiectasia mutated; CCND1/D2/E1, cyclin D1/D2/E1; CDK6, cyclin-dependent kinase 6; CDKN1A, cyclin-dependent kinase inhibitor 1A; GADD45A/B/G, growth arrest and DNA damage-inducible protein 45A/B/G; IGFBP3, insulin-like growth factor-binding protein 3; MMGT1, membrane magnesium transporter 1; PMAIP1, phorbol-12-myristate-13-acetate-induced protein 1; SESN2, sestrin-2; STEAP3 (=TSAP6), STEAP3 metalloreductase; ZMAT3 (=PAG608), zinc finger matrin-type 3.

**Table 2:** PCR primer sequences.

Gene		Position	Sequence	Product Length
*SESN2* (NM_031459.5)	sense	1455-1475	5′-CAATACCATCGCCATGCACAG-3′	246 bp
	antisense	1700-1680	5′-AAGTTCACGTGGACCTTCTCT-3‘	
*HPRT* (NM_000194.3)	sense	371-390	5’-TTGCTGACCTGCTGGATTAC-3’	262 bp
	antisense	632-613	5’-CTTGCGACCTTGACCATCTT-3’	

SESN2, Sestrin-2; HPRT, hypoxanthine-guanine phosphoribosyltransferase; bp, base pair.

**Table 3:** Induced KEGG pathways in breast cancer cells under hypoxia compared to normoxia (MCF-7: column 1 and MDA-MB-231: column 4), as well as after treatment with 10 µM BA compared to DMSO treatment under normoxia (MCF-7: column 2 and MDA-MB-231: column 5) or hypoxia (MCF-7: column 3 and MDA-MB-231: column 6). Red label corresponds to significant regulated KEGG pathways (Benjamini-Hocberg adjusted *p* < 0.05).

	MCF-7 (wtp53)			MDA-MB-231 (mtp53)		
	DMSO	DMSO vs. BA		DMSO	DMSO vs. BA	
KEGG Pathway	Normoxia vs. Hypoxia	Normoxia	Hypoxia	Normoxia vs. Hypoxia	Normoxia	Hypoxia
hsa00010:Glycolysis/Gluconeogenesis						
hsa00051:Fructose and mannose metabolism						
hsa00230:Purine metabolism						
hsa00240:Pyrimidine metabolism						
hsa00280:Valine, leucine and isoleucine degradation						
hsa00620:Pyruvate metabolism						
hsa00640:Propanoate metabolism						
hsa00670:One carbon pool by folate						
hsa00970:Aminoacyl-tRNA biosynthesis						
hsa03030:DNA replication						
hsa03040:Spliceosome						
hsa03410:Base excision repair						
hsa03430:Mismatch repair						
hsa04110:Cell cycle						
hsa04115:p53 signaling pathway						
hsa04120:Ubiquitin mediated proteolysis						
hsa04142:Lysosome						
hsa04510:Focal adhesion						
hsa04621:NOD-like receptor signaling pathway						
hsa04722:Neurotrophin signaling pathway						
hsa05200:Pathways in cancer						
hsa05222:Small cell lung cancer						

**Table 4:** Log2-fold-change of gene expression caused by BA treatment referred to DMSO-treated cells.

	MDA-MB-231		HS578T		T47D		MCF-7		Cal51		
Gene	Normoxia	Hypoxia	Normoxia	Hypoxia	Normoxia	Hypoxia	Normoxia	Hypoxia	Normoxia	Hypoxia	Gene
*ATM*	−0.2	−0.4	n.d.	n.d.	n.d.	n.d.	−0.1	−0.5	n.d.	n.d.	*ATM*
*CCND1*	−0.1	−0.7 **	−0.1	0.6 **	0.3 *	1.0 *	−0.3 **	−0.3	−0.1	−0.1	*CCND1*
*CCND2*	1.4 **	−0.3 *	n.v.	n.v.	n.v.	n.v.	1.3	−0.9	−0.3	−0.4	*CCND2*
*CCNE1*	0.1	1.1 ***	0.3	0.4	−0.3 **	1.5 **	−0.3	0.9 *	−0.3	−0.3 *	*CCNE1*
*CDK6*	0.2	0	0.1	0.5	0.6	0.0	0.2	−1.0 **	−0.1	0.1	*CDK6*
*CDKN1A*	1.3 *	1.6 ***	0.1	0.2	0.4	1.5 **	0.6	0.0	−0.1	0.2	*CDKN1A*
*GADD45A*	2.1 *	2.5 **	0.6	0.5	1.9 **	1.4 ***	1.7	0.6	0.4	1.3 *	*GADD45A*
*GADD45B*	0.2 **	1.3 ***	0.2	−0.4	0.3 *	1.3 *	0.1	0.1	0.0	0.6 *	*GADD45B*
*GADD45G*	1,8 **	4.3 ***	n.d.	n.d.	1.5 **	1.6 *	0.8 **	0.4	0.5	0.3	*GADD45G*
*IGFBP3*	−0.2	−0.8 ***	0.8 *	−0.5	1.1 **	1.2 *	0.5	0.6	−0.2	0.2	*IGFBP3*
*PMAIP1*	1.4 **	1.3 ***	0.4 *	0.5	1.0 ***	2.5 ***	0.5	0.5	0.4	1.0 **	*PMAIP1*
*SESN2*	2.5 **	2.2 ***	1.0	0.2	1.2 **	1.8	1.1 *	1.8 *	0.4	0.8 *	*SESN2*
*STEAP3*	−0.4 ***	−0.9 ***	0.1	−1.0 **	0.2 *	0.2	0.0	0.0	0.0	0.1	*STEAP3*
*ZMAT3*	0.2	0.1	n.d.	n.d.	n.d.	n.d.	0.3	-0.4	n.d.	n.d.	*ZMAT3*

ATM, ataxia telangiectasia mutated; CCND1/D2/E1, cyclin D1/D2/E1; CDK6, cyclin-dependent kinase 6; CDKN1A, cyclin-dependent kinase inhibitor 1A; GADD45A/B/G, growth arrest and DNA damage-inducible protein 45A/B/G; IGFBP3, insulin-like growth factor-binding protein 3; PMAIP1, phorbol-12-myristate-13-acetate-induced protein 1; SESN2, sestrin-2; STEAP3 (=TSAP6), STEAP3 metalloreductase; ZMAT3 (=PAG608), zinc finger matrin-type 3; n.d., not determined; n.v., not verifiable; * ≤0.05; ** ≤0.01; *** ≤0.001.

## Data Availability

Not applicable.

## Supplementary Materials

The following supporting information can be downloaded at: https://www.mdpi.com/article/10.3390/cells12010177/s1, Figure S1: Principal component analysis (PCA) blot of microarray data; Figure S2: Comparison of log2-fold-change of qRT-PCR and microarray data; Table S1: log2-fold-change of gene expression caused by hypoxia referred to normoxia.
