# Supplementary material for: SESN2 Knockdown Increases Betulinic Acid-Induced Radiosensitivity of Hypoxic Breast Cancer Cells
Source: Cells. 2022 Dec 31;12(1):177. doi: 10.3390/cells12010177 (PMC9818433; doi:10.3390/cells12010177)
Supplement: Supplementary file 1 [file cells-12-00177-s001.zip › Cells_Supplementary.pdf]

Supplementary Files

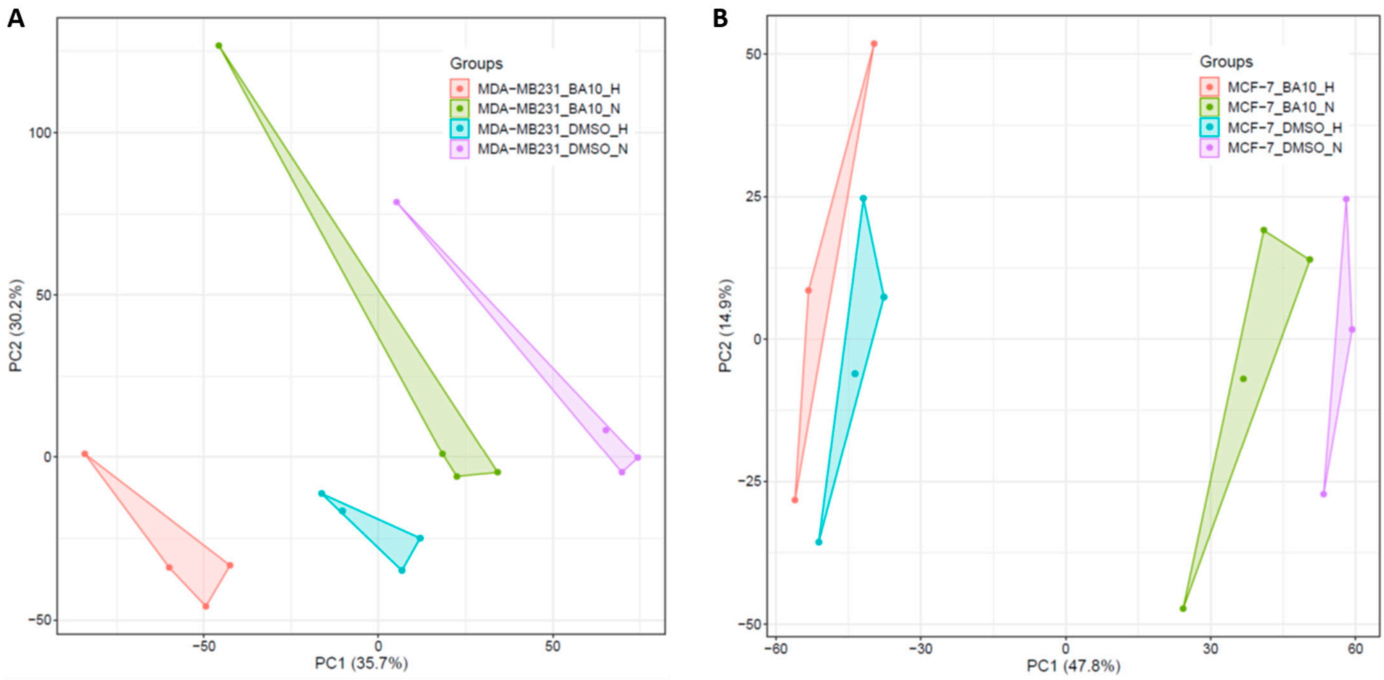

Supplementary Figure S1: Principal component analysis (PCA) blot of microarray data

PCA based on the normalized gene expression profile of BS or DMSO treatment under normoxic or hypoxic conditions in MDA-MB-231 (A) or MCF-7 (B) cells. PCA was calculated using data from three or four independent experiments. Principal component analysis (PCA) of the microarray dataset revealed, that the main effects were caused by the different oxygen level (normoxia vs. hypoxia). The effects of 10 µM BA were less pronounced.

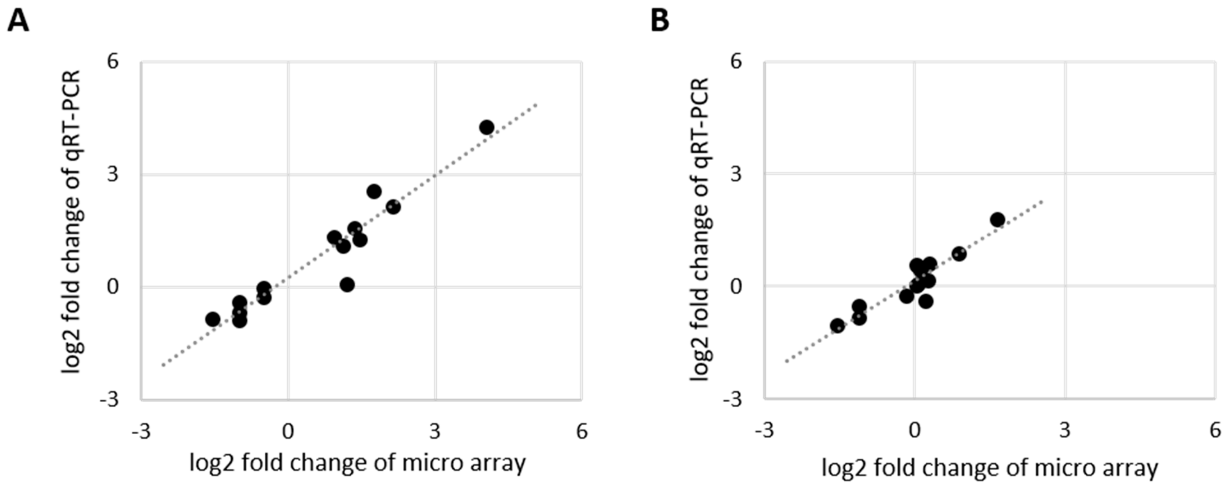

Supplementary Figure S2: Comparison of log2-fold-change of qRT-PCR and microarray data

MDA-MB-231 (A) and MCF-7 (B) cells were treated with 10 µM BA for 48 h. Log2-fold-change of fourteen differentially expressed genes was determined in BA-treated cells compared to DMSO-treated cells measured by qRT-PCR. Data were fitted to a linear regression model (dotted line). Data represent mean values of at least three independent experiments.

In MDA-MB-231 cells we calculated a correlation coefficient R of 0.95 and in MCF-7 cells of 0.92.

Supplementary Table S1: log2-fold-change of gene expression caused by hypoxia referred to normoxia

gene	MDA-MB-231	HS578T	T47D	MCF-7	Cal51	gene
ATM	0.2	n.d.	n.d.	0.9 **	n.d.	*ATM*
CCND1	-0.4 **	0.1	-0.7 ***	0.2	0.7 **	*CCND1*
CCND2	3.0 ***	n.v.	n.v.	2.5	2.8 **	*CCND2*
CCNE1	-0.2	0.5	-2.1 ***	-1.3	-0.6 *	*CCNE1*
CDK6	0.4	1.1 **	0.3	0.5 *	0.4 **	*CDK6*
CDKN1A	1.7 ***	3.4 **	1.6 **	1.7 ***	1.2 ***	*CDKN1A*
GADD45A	0.5	1.4 **	4.5 ***	2.3 *	2.4 *	*GADD45A*
GADD45B	0.4 ***	3.1 ***	1.8 **	1.5 ***	1.3 **	*GADD45B*
GADD45G	3.2 ***	n.d.	3.6 ***	0.2	3.9 **	*GADD45G*
IGFBP3	2.9 ***	6.1 ***	4.6 ***	4.7 ***	1.7 **	*IGFBP3*
PMAIP1	0.5 ***	1.8 ***	2.7 ***	0.0	1.1 **	*PMAIP1*
SESN2	1.6 ***	3.8 ***	1.9 ***	1.2 **	0.8 *	*SESN2*
STEAP3	-0.1	2.0 ***	-0.8 ***	0.0	-0.6 **	*STEAP3*
ZMAT3	0.5 **	n.d.	n.d.	0.7 **	n.d.	*ZMAT3*

ATM, ataxia telangiectasia mutated; CCND1/D2/E1, cyclin D1/D2/E1; CDK6, cyclin-dependent kinase 6; CDKN1A, cyclin-dependent kinase inhibitor 1A; GADD45A/B/G, growth arrest and DNA damage-inducible protein 45A/B/G; IGFBP3, insulin-like growth factor-binding protein 3; PMAIP1, phorbol-12-myristate-13-acetate-induced protein 1; SESN2, sestrin-2; STEAP3, STEAP3 metalloreductase; ZMAT3 (=PAG608), zinc finger matrin-type 3; n.d., not determined; n.v., not verifiable; * ≤ 0.05; ** ≤ 0.01; *** ≤ 0.001
