# Supplementary figures and images for: SESN2 Knockdown Increases Betulinic Acid-Induced Radiosensitivity of Hypoxic Breast Cancer Cells
Source: Cells. 2022 Dec 31;12(1):177. doi: 10.3390/cells12010177 (PMC9818433; doi:10.3390/cells12010177)

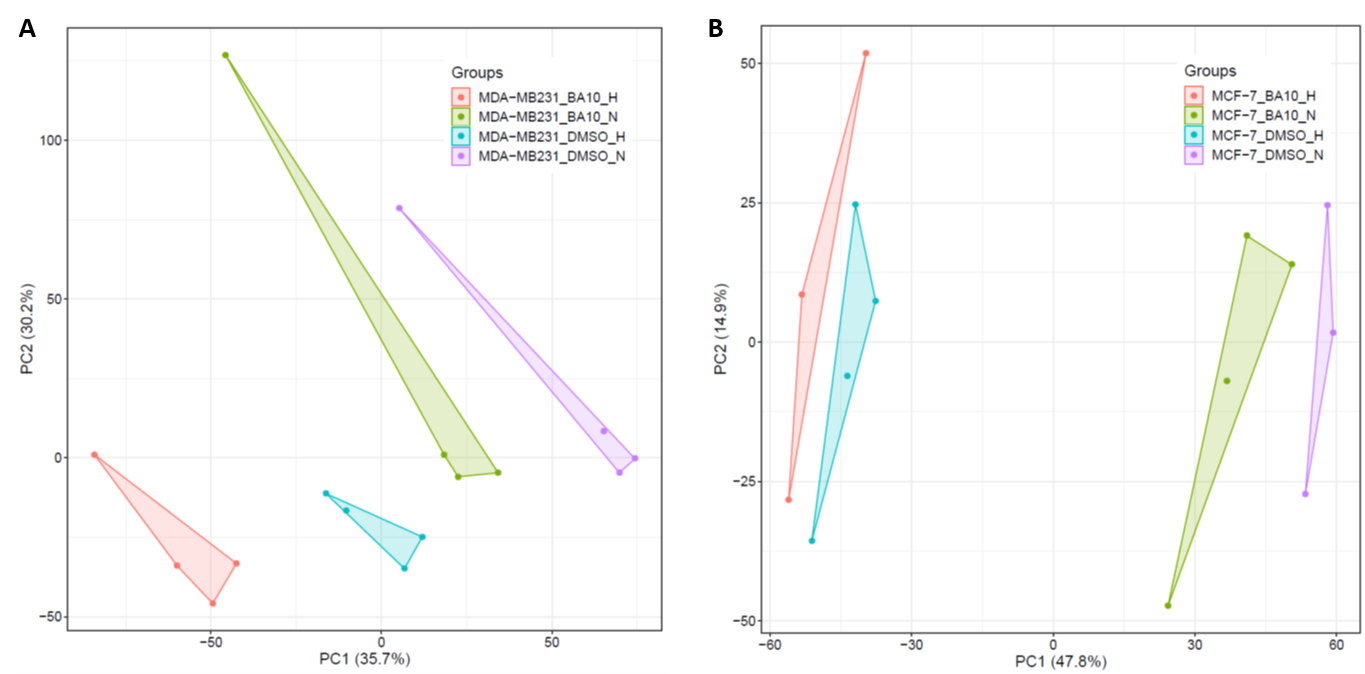

Supplement: Supplementary file 1 [file cells-12-00177-s001.zip › Supp.Figure 1 final.tif]

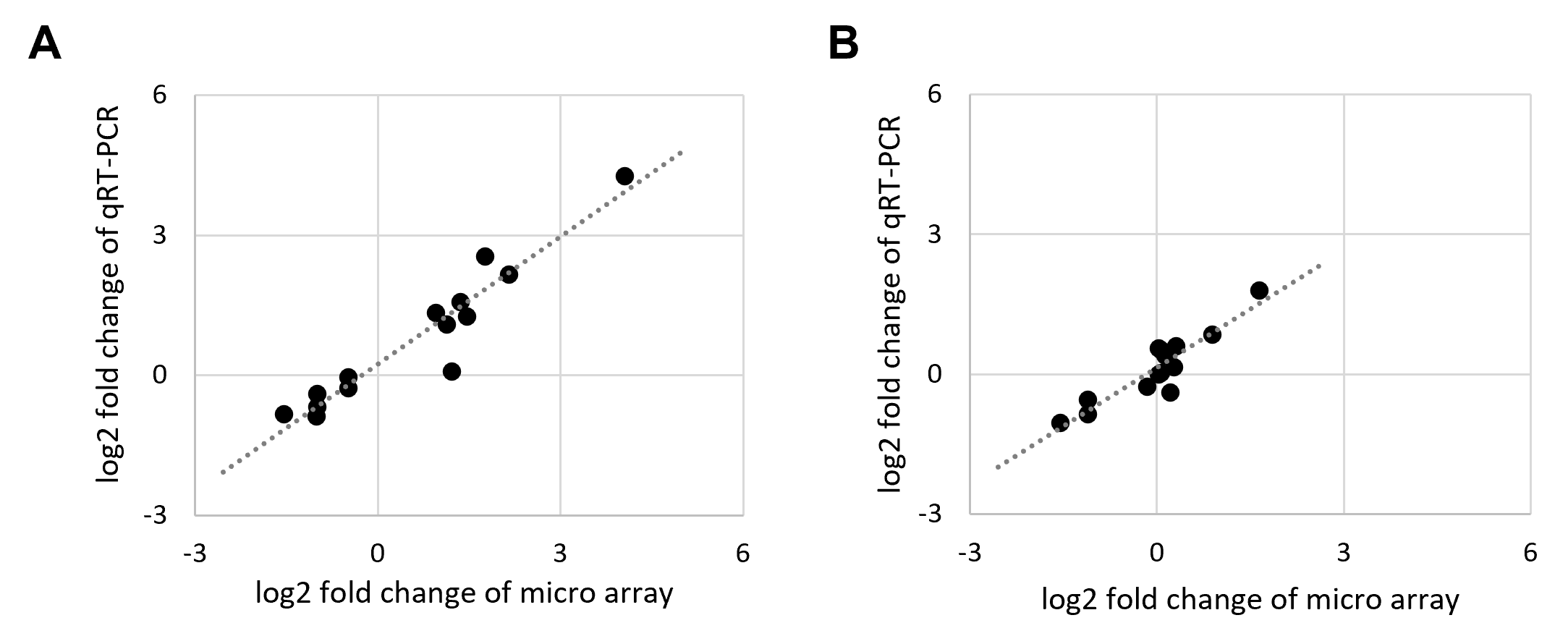

Supplement: Supplementary file 1 [file cells-12-00177-s001.zip › Supp.Figure 2 final.tif]
